# Prediction of post-operative necrosis after mastectomy: A pilot study utilizing optical diffusion imaging spectroscopy

**DOI:** 10.1186/1477-7819-7-91

**Published:** 2009-11-25

**Authors:** Roshni Rao, Michel Saint-Cyr, Aye Moe Thu Ma, Monet Bowling, Daniel A Hatef, Valerie Andrews, Xian-Jin Xie, Theresa Zogakis, Rod Rohrich

**Affiliations:** 1 Department of Surgery, Division of Surgical Oncology, University of Texas, Southwestern Medical Center, 5323 Harry Hines Blvd, Dallas, TX 75390-9155, USA; 2 Department of Plastic Surgery, University of Texas Southwestern Medical Center, 5323 Harry Hines Blvd., Dallas, TX 75390-9155, USA; 3 Department of Clinical Sciences-Division of Biostatistics, University of Texas Southwestern Medical Center, 5323 Harry Hines Blvd, Dallas, TX 75390-9155, USA

## Abstract

**Introduction:**

Flap necrosis and epidermolysis occurs in 18-30% of all mastectomies. Complications may be prevented by intra-operative detection of ischemia. Currently, no technique enables quantitative valuation of mastectomy skin perfusion. Optical Diffusion Imaging Spectroscopy (ViOptix T.Ox Tissue Oximeter) measures the ratio of oxyhemoglobin to deoxyhemoglobin over a 1 × 1 cm area to obtain a non-invasive measurement of perfusion (StO_2_).

**Methods:**

This study evaluates the ability of ViOptix T.Ox Tissue Oximeter to predict mastectomy flap necrosis. StO_2 _measurements were taken at five points before and at completion of dissection in 10 patients. Data collected included: demographics, tumor size, flap length/thickness, co-morbidities, procedure length, and wound complications.

**Results:**

One patient experienced mastectomy skin flap necrosis. Five patients underwent immediate reconstruction, including the patient with necrosis. Statistically significant factors contributing to necrosis included reduction in medial flap StO_2 _(p = 0.0189), reduction in inferior flap StO_2 _(p = 0.003), and flap length (p = 0.009).

**Conclusion:**

StO_2 _reductions may be utilized to identify impaired perfusion in mastectomy skin flaps.

## Synopsis

In this pilot study of ten patients, increased mastectomy flap length, a significant drop in medial and inferior StO_2 _measurements by Optical Diffusion Imaging Spectroscopy (ViOptix T.Ox Tissue Oximeter) intra-operatively predicted post-operative mastectomy skin flap necrosis.

## Introduction

Breast cancer is diagnosed in approximately 200,000 women in the United States every year. Surgical treatment for breast cancer involves either breast conserving surgery (BCT) or total mastectomy. Although recent studies [1] indicate that the majority of patients diagnosed with breast cancer receive BCT, 33% of patients continue to undergo mastectomy [1]. There also appears to be a significant improvement in the utilization of post-mastectomy reconstruction across the country [2]. Although the benefits of immediate reconstruction after mastectomy are well-documented [3], it has also been demonstrated that immediate reconstruction does increase the rate of post-operative wound complications [4]. Wound complications following mastectomy are estimated to be between 18-30% [5,6]. Common complications include partial flap necrosis, epidermolysis and eschar formation.

Overall cosmetic outcome is highly dependent on the viability of mastectomy skin flaps. There is currently no accepted standard for evaluating skin flaps in the intra-operative setting. Techniques which are utilized include the injection of fluorescein, evaluation of "bleeding edges", and subjective assessment of capillary refill. Near Infrared Spectroscopy is a non-invasive method used to monitor blood perfusion to skin flaps. The unit of measurement is StO_2_. This is a measurement of the ratio of oxyhemoglobin (HgbO_2_) and deoxyhemoglobin (Hgb) in order to obtain noninvasive, real-time measurement of tissue pO_2_. This technique has previously been validated and is commonly used by plastic and reconstructive surgeons to assess the perfusion and viability of donor digital implants and microsurgical free tissue transfers [7-9]. The current pilot study evaluates the ability of near infrared spectroscopy to predict post-mastectomy skin flap necrosis in 10 patients.

## Methods

Approval for the protocol was obtained from the Institutional Review Board at the University of Texas Southwestern Medical Center. Ten patients undergoing mastectomy at a single institution were selected for the study. Data recorded included patient age, height/weight, co-morbidities, smoking history, medical history, tumor size, pathology and stage.

### Tissue Oximeter

The ViOptix T.Ox Tissue Oximeter Tissue Oximeter^® ^made by ViOptix, Inc. (Fremont, CA) was used to obtain tissue oxygen saturation (StO_2_) measurements. Near-infrared lights of 690-nm and 830-nm wavelengths are emitted at a scan rate of up to 40 Hz and are transmitted to the tissue through a special quartz fiberglass cable. The light is absorbed, scattered, and reflected in the layers of the tissue up to 10 mm deep, including the capillary loops and dermal plexus. The light is absorbed by biological compounds known as chromophores, whose absorption properties are oxygen-dependent. Common chromophores include hemoglobin, myoglobin, and cytochrome c oxidase. The volume of tissue under investigation is determined by the depth of near infrared light penetration (10 mm). The amount of light recovered from tissues is dependent on the intensity of incident light, separation of the optodes, degree of light scattering in tissues, and amount of absorption by chromophores. Since the intensity, distance between the optodes and light scattering are controlled, the changes in recovered light can be attributed to the variation in the concentration of chromophores. The recovered light is then processed by an integrated computer performing a fingerprint analysis of the spectral data. The data is then displayed in real-time, numerically, on a monitor.

### Patients

**Figure 1:**
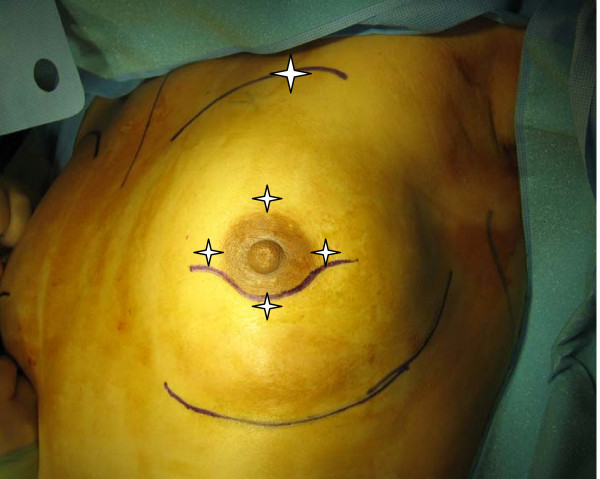
**Cardinal points of measurement pre-operatively**.

A cohort of patients was selected who were undergoing mastectomy--both skin-sparing and traditional mastectomy patients were chosen to more accurately reflect the heterogeneity encountered by the practicing surgeon. Measurements were made preoperatively, and immediately after dissection at the following locations: superior mastectomy skin flap; lateral mastectomy skin flap; medial mastectomy skin flap; inferior mastectomy skin flap; and 2 cm inferior to the clavicle (Figure 1). Method of reconstruction, mastectomy operative time, measurements of the thickness of each skin flap, and length from clavicle to superior edge of the mastectomy skin flap were all recorded.

### Measurements

Flap thickness was measured by allowing the skin to lie in a neutral position against the chest wall and then utilizing an intra-operative ruler to measure the skin flap at its most distal aspect. Flap length was defined as the superior flap length, this area was measured since this is typically the longest flap in a mastectomy. It was measured by allowing all skin to lie in a neutral position and measuring the distance, in cm, from the edge of the superior portion of the incision at the 12 o'clock position to the clavicle, care was taken to ensure that a straight line was maintained during this measurement. All complications were noted; presence and total area of epidermolysis was noted and recorded. Patients were followed for four weeks post-operatively to estimate the area of necrosis, evaluate for wound infection, and seroma formation. De-identified data was entered into a Microsoft Excel^® ^database. Statistical analysis was performed using Wilcoxon Rank Sum test and Student's *t*-test.

## Results

Of the 10 patients in this study, 1 (10%) developed significant mastectomy skin flap necrosis. Measurements were obtained during the operation, the first one just prior to dissection, and the 2^nd ^at the completion of the mastectomy, comparisons were then performed between these numbers. Statistically significant factors predicting post-op necrosis included reductions in medial (p = 0.0189) and inferior (p = 0.003) StO_2 _levels, and flap length (p = 0.009) (Table 1). In the patient who experienced necrosis, medial StO_2 _reduction was 61% (p = 0.049), corresponding with an absolute medial StO_2 _reduction of 42 points. Patients who did not have necrosis actually had an increase in their medial StO_2 _of 14.6%, corresponding with an absolute medial StO_2 _increase of 6.7 points. The patient with necrosis had a 69% decrease in inferior StO_2 _levels, corresponding with a 65.5 point drop (p = 0.003). Patients without necrosis demonstrated a 20% increase in inferior StO_2 _levels, corresponding with a 9.8 point increase in absolute StO_2 _levels. The patient with necrosis had a 15 cm flap length, as opposed to a 11.9 cm average flap length in the other 9 patients (p = 0.009).

**Table 1:** Analysis of patients with and without necrosis

	Necrosis		
		
	Yes (1)	No (9)	p-value
**Age**	57	48	0.278
**Seroma**	0	1	
**Infection**	0	1	
**Diabetes**	0	2	
**Radiation**	0	1	
**Hypertension**	1	4	
			
***Flap Length (cm)***	***15***	***11.9***	***0.009***
			
**Thickness of flap (mm)**			
Superior	3.0	4.2	0.201
Inferior	3.0	4.4	0.100
Lateral	4.0	40	1.000
Medial	4.0	4.0	1.000
			
**Pre-operative Tissue Oxygenation (StO2)**			
Superior	59.0	60.9	0.910
***Inferior***	***94.0***	***49.1***	***0.0017***
Lateral	73.5	58.2	0.371
Medial	68.5	58.5	0.540
			
**Post-operative Tissue Oxygenation (StO2)**			
Superior	28	54.4	0.083
Inferior	29	59.0	0.199
Lateral	50	62.2	.0586
Medial	27	65.2	0.058
			
**Changes in Tissue Oxygenation** **StO2 percent change (absolute StO2 change)**			
Superior	-53% (-31.5)	-5.9% (-6.5)	0.280
***Inferior***	***-69% (-65.5)***	***+20% (+9.8)***	***0.003***
Lateral	-32% (-23.5)	+7.17% (+4.1)	0.145
***Medial***	***-61% (-42)***	***+14.6% (+6.7)***	***0.018***
Clavicular	+6% (+2)	+14.6% (+6.8)	0.850

Variables analyzed, statistically significant variables are bold and italicized

Patient demographics are displayed in Table 2. Fifty percent of patients were African-American, 40% were Hispanic, 10% were White. The average age was 49, average body mass index (BMI) was 27.9. There were two patients with diabetes and five with hypertension. None of the patients had chronic obstructive pulmonary disease (COPD) or admitted to smoking. Only one patient had evidence of tumor skin involvement. The stage of the primary tumor ranged from DCIS to T4D. Three patients had DCIS, and five had invasive ductal cancer. Five patients had undergone neoadjuvant chemotherapy, and one had previously received radiation to the chest wall. Average operative time was 109 minutes (60-180 min), a factor which was not significantly different between the two groups. The one patient with necrosis did have an expander in place, four of the patients without necrosis also had expanders, all of these patients underwent skin-sparing mastectomy. There were no nipple-sparing mastectomies in this cohort. The remaining five patients did not undergo immediate reconstruction and underwent mastectomy with a standard elliptical incision. Operative time, BMI, tumor pathology, tumor size, patient age and operating surgeon were not significant factors in predicting necrosis.

**Table 2:** Patient Demographics

Factor	% (n)
**Average Age**	49
	
**Race**	
White	10% (1)
African American	50% (5)
Hispanic	40% (4)
	
**Body Mass Index (BMI)**	
Average	27.9
	
**Smoking**	0% (0)
	
**Co-morbidities**	
Diabetes	20% (2)
Hypertension	50% (5)
COPD	0% (0)
	
**Skin Involvement**	
None	80% (8)
Skin retraction	10% (1)
	
**Clinical T Size**	
Tis	30% (3)
T0	10% (1)
T1	30% (3)
T3	10% (1)
T4A	10% (1)
T4D	10% (1)
	
**Histology**	
DCIS	30% (3)
Invasive Ductal	50% (5)
Invasive Lobular	10% (1)
Other	10% (1)
	
**Neoadjuvant Chemo**	50% (5)
	
**Radiation to Chest Wall**	10%(10)

**Figure 2:**
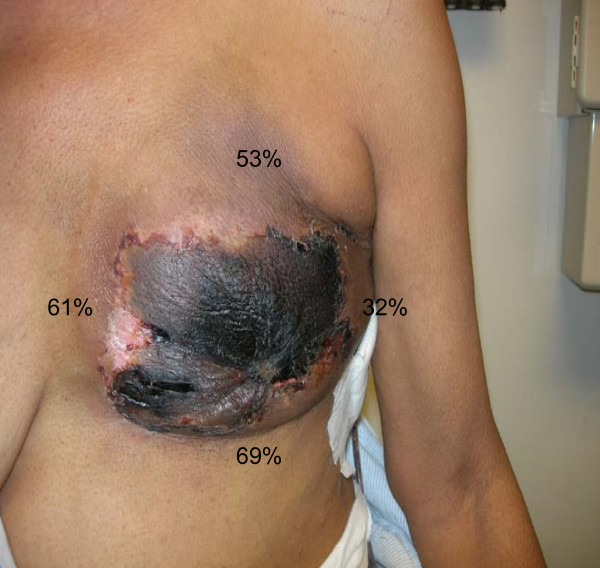
**A patient with significant intraoperative decrease in StO_2_**. Decreases were: 53%, 69%, 61%, and 32% at superior, inferior, medial, and lateral, respectively.

The patient with 108 cm^2 ^of necrosis (Figure 2) underwent skin-sparing mastectomy, sentinel node biopsy and immediate reconstruction with expander placement. The expander was not filled intra-operatively. This patient had uniquely significant drops in StO_2 _measurements post-operatively (Figure 2). This patient had full thickness necrosis in several areas of the mastectomy skin flap. She did have a personal history of Hepatitis C, sarcoidosis, and hypertension. Intraoperative fluorescein dye injection was also used to assess mastectomy skin flap viability and did indicate a possible perfusion deficit at the 2 o' clock position. Due to the overlying skin necrosis and consequent exposed expander, she required expander removal and skin graft two months after her mastectomy.

## Discussion

One commonly used tool to evaluate mastectomy flap viability intra-operatively is the intravenous sodium fluorescein test (Wood's lamp method). This involves intravenous injection of fluorescein followed by intra-operative evaluation with a Wood's lamp. Although it has been available since 1931, its application is prone to subjective errors, and is limited to over/under reading by as much as 30% [10]. It is also a test of vascularity - not viability, and subject to changes in vascularity such as vasospasm, intravascular clotting, or alterations in the distribution of the microcirculation. Alternatively the use of infrared spectroscopy takes into account metabolic changes of the dissected tissue, and potentially allows trends to be followed for flap evaluation post-operatively.

The arterial supply of the breast is generally defined as an anastomotic plexus of vessels originating from the axillary artery, the internal mammary artery, the intercostal arteries, and lateral thoracic artery. The contribution of each individual artery and the consequences of vascular interruption are poorly understood, but the course of the nerves and vessels may be related to the ligamentous apparatus [11]. One such horizontal ligamentous suspension originates from the pectoral fascia along the 5^th ^rib [12]. Our finding that the decrease in perfusion from the inferior portion of the breast most accurately predicted post-operative epidermolysis may be supportive of this finding.

In addition, there currently does not exist any standardized method for measuring mastectomy skin flap thickness during an operation, further refinements in this technique-i.e. the use of calipers, may be helpful for future trials.

Traditionally, surgeons are careful to avoid transection of medial perforators. Consistent with this, our data demonstrate an increased likelihood of necrosis in the patient who had a significant decrease in medial StO_2 _measurements. This may be particularly important in those patients who undergo disruption of the medial perforators secondary to internal mammary node dissection.

There are significant limitations to this study. Most notable is the small sample size. Contributions from underlying co-morbidities (coronary artery disease, diabetes) may be more readily apparent with a larger sample size. In addition, this study population was predominately a minority population; there is an under-representation of Caucasian patients. Although the ViOptix T.Ox Tissue Oximeter system has been validated in several racial groups, there may be variability in StO_2 _measurements between races which can only be further elucidated with a large sample size. For further studies, assuming a 10% necrosis rate, a sample of 40 patients will provide more than 90% power to detect a two standard deviation difference of the mean StO_2 _measures (significance level is held at 0.05, two sided). Clearly a group of patients undergoing skin-sparing mastectomy with immediate reconstruction would provide the most useful clinical information as these patients are more likely to have difficulties with wound healing and face the greatest consequences (implant extrusion, flap failure) from poor wound healing.

It is known that the perfusion to the subdermal plexus of the skin is controlled by the autonomic nervous system in response to variations in metabolic demands and environment. All patients in this study were stable intra-operatively. However, the actual oxygen saturation and blood pressure measurements at the time of StO_2 _measurement were not evaluated, the influence of these factors will be examined in future studies. The patient with necrosis had drops in StO_2 _measurement, which also may be an indicator of failure to compensate for injury, whereas the patients who did not have necrosis, for the most part, had increased StO_2 _levels after dissection, potentially indicating an ability to increase perfusion appropriately to the area of injury.

Similarly, wound healing is a complicated process. Factors contributing to or complicating the wound healing process include body habitus, age, co-morbidities, prolonged operative time, collagen disorders, infection, history of radiation exposure, immune status, and steroid use [13-15].

Lastly, a review of the patient response to the ViOptix T.Ox Tissue Oximeter system indicates that the patient having necrosis also had a longer flap length. This would appear to be consistent with the concept that the blood supply of longer flaps is more tenuous, likely due to the greater area of vascular disruption required when a mastectomy is performed.

## Conclusion

Commonly used intraoperative methods to determine flap viability include detection of skin discoloration, wound edge bleeding and intra-operative assessment with fluorescein and a Wood's Lamp. The use of near-infrared reflection spectroscopy to monitor myocutaneous flaps has been previously validated in humans [9]. Our study indicates that ViOptix T.Ox Tissue Oximeter is a non-invasive method which may be utilized to identify impaired perfusion in mastectomy skin flaps. It could potentially add valuable information to clinical observation, and may be able to detect early vascular complications. Areas which demonstrate sub-optimal perfusion can therefore be excised intra-operatively to potentially decrease wound complications and improve cosmetic outcome, alternatively, reconstruction may also be postponed until a later date or potentially an autologous reconstruction may be considered. Further studies are planned with a larger sample size for validation, and to establish standards.

## Consent

Written informed consent was obtained from the patient for publication of this case report and accompanying images. A copy of the written consent is available for review by the Editor-In-Chief of this journal.

## Competing interests

The authors declare that they have no competing interests.

## Authors' contributions

RR initiated this research & enrolled patients, & wrote the initial manuscript, MS-C designed the study, assisted with writing the manuscript & enrolled patients, AMTM collected data and wrote portions of the manuscript, MB collected data, DH collected data and assisted with study design, VA enrolled patients and performed measurements, X-JX performed all statistical analysis, TZ enrolled patients and performed measurements, RR enrolled patients and assisted with manuscript writing. All authors have read and approved the final manuscript.
